# Surgical breast cancer patient pathway: Experiences of patients and relatives and their unmet needs

**DOI:** 10.1111/hex.12869

**Published:** 2019-01-12

**Authors:** Ingrid Annette Zøylner, Kirsten Lomborg, Peer Michael Christiansen, Pia Kirkegaard

**Affiliations:** ^1^ Department of Plastic and Breast Surgery Aarhus University Hospital Aarhus Denmark; ^2^ Department of Clinical Medicine Aarhus University Aarhus Denmark; ^3^ Department of Surgery Randers Regional Hospital Randers Denmark; ^4^ Department of Public Health Programmes Randers Regional Hospital Randers Denmark

**Keywords:** breast neoplasms, cancer patient pathway, focus groups, health service research, patient experience, patient participation

## Abstract

**Background and aim:**

Breast cancer is the most common cancer disease in women worldwide. In Denmark, the law prescribes cancer patient pathways (CPPs) in general and thus also for breast cancer. Although results from patient satisfaction surveys show overall satisfaction with the pathway, a call for improvement has been voiced for some areas. The aim of this study was to explore patients’ and relatives’ experiences with the surgical breast CPP and to identify any unmet needs.

**Method:**

This study was based on focus groups with patients who had surgery for breast cancer, and their relatives. The settings were two Danish surgical breast cancer clinics.

**Findings:**

Overall, patients and relatives found the structure of the surgical breast CPP satisfactory. The time in the surgical department was short, and most patients found it difficult to cope with the situation. Empathy and a supportive relationship between patients, relatives and health‐care professionals were of great importance. Five key points were identified in which some of the participants had unmet needs. Suggestions for change were related to information, communication, choice of treatment, flexibility in the pathway and easy access to the clinic after surgery.

**Conclusion:**

Although patients and relatives found the CPP for breast cancer satisfactory and well planned, suggestions for change were made relating to unmet needs with respect to five key points in the pathway. Implementing findings from this study in clinical practice requires co‐operation between health‐care professionals and support from the leaders of the organization.

## 1 BACKGROUND

Breast cancer is the most common cancer disease in women in Western countries, and the diagnostic period is stressful for most breast cancer patients due to fear of possible implications of the treatment and fear of dying.^1^, ^2^, ^3^, ^4^ A systematic review on psychosocial and practical support needs has suggested that cancer patients, including patients with breast cancer, experience unmet needs related to physical and psychosocial aspects at the time of diagnosis.^5^ The time between diagnosis and surgery is of special concern, and it has been suggested that health‐care professionals (HCPs) offer individual support and information during this period.^6^

In Denmark, approximately one in every nine women gets breast cancer, and most of them are between 50 and 70 years old.^7^ The survival rate has increased in recent decades^8^ and is 97% after 1 year and 87% after 5 years.^9^ The Danish health authorities stipulate cancer patient pathways (CPP)s for cancer treatment.^10^ Patients may access the breast CPP via their general practitioner (GP) or via mammography screening. If a patient has surgery, she will receive the surgery results as a part of the CPP.^10^

The majority of breast cancer patients undergo day surgery; about 70% have breast‐conserving surgery and 30% have mastectomy. Furthermore, most patients are offered adjuvant therapy.^11^, ^12^ Day surgery for breast cancer appears to be safe and well tolerated with good satisfaction rates.^13^, ^14^ However, short duration of time generally affects the ability to absorb information^15^ and there has been a call for better ways to offer emotional support, counselling and information about the disease and its management.^14^ Results from the Danish National Patient Satisfaction Surveys^16^, ^17^ showed high overall satisfaction, but low satisfaction was related to patients’ and relatives’ experiences of being involved in treatment and health‐care decisions, the amount of information received and knowledge about who among the HCPs are responsible for their treatment course. However, there is little evidence that data on patient satisfaction lead to improvements in the quality of care.^18^ Many factors are modifying the association between the received care and patient‐reported satisfaction, for example, patient expectations, patient characteristics and loyalty to HCPs.^19^ In addition, patients tend to answer more positively to general questions about their overall experience despite having reported critical events, and patients tend to be more critical if they have the possibility to explain their criticism (ibid). It makes it difficult for HCPs to act on behalf of the patient satisfaction surveys alone. Thus, deeper insight into patients with breast cancer and their relatives’ experiences of the surgical CPP for breast cancer is needed.

The aim of this study was to explore patients’ and relatives’ experiences with the surgical breast CPP and to identify any unmet needs.

## 2 METHODS

The study draws on qualitative focus groups (FGs)^20^ with patients treated for breast cancer and their relatives. It is the first part of an action research study aiming at involving patients with breast cancer and their relatives in the development of the surgical breast CPP. The action research approach leans towards the pragmatic philosophy of John Dewey^21^ focusing on people's active involvement in making sense of their world.

### 2.1 Setting

**Figure 1:**
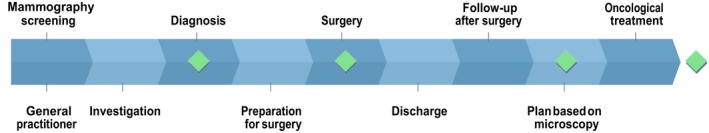
Surgical breast cancer patient pathway . 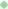 Key points in the surgical breast cancer patient pathway based on scientific literature^1^, ^6^, ^22^, ^23^, ^24^, ^25^, ^26^, ^27^, ^28^, ^29^ and confirmed by healthcare professionals experience

Two surgical breast cancer clinics in Denmark were involved. Both clinics used the same surgical guidelines^11^ and CPP.^10^ During the recruitment period, 556 patients had surgery for breast cancer in the clinics. Figure 1 illustrates the surgical breast CPP. “Key points” identify events of special importance for patients with breast cancer derived from scientific literature^1^, ^6^, ^22^, ^23^, ^24^, ^25^, ^26^, ^27^, ^28^, ^29^ and were confirmed by the HCPs. The key points were as follows: receiving the diagnosis; the day of surgery; plan for further treatment based on microscopy; after completing overall treatment.

### 2.2 Participants and recruitment

Former patients who had surgery for breast cancer were identified through the Danish Breast Cancer Cooperative Group database. We invited patients with a temporal distance to the surgery, because we did not want to burden the patients during their adjuvant treatment. We selected civil registration numbers starting from 01.01 and continued until we had included 100 patients from each clinic. Three to five FGs with five to eight participants in each setting are recommended in the literature.^20^, ^30^ Based on a report on Danes’ feedback to the health‐care system,^31^ we assumed that 30%‐40% of the patients would respond to the invitation.

We excluded patients with more advanced breast cancer (Stage III and IV), bilateral breast cancer, patients treated with neo‐adjuvant chemotherapy and male patients because they do not follow the standard surgical breast CPP (Figure 1).

The patients received letters with an information sheet, a written consent form (Appendix S1) and prepaid, pre‐addressed envelopes. The form allowed them to accept or reject participation and to indicate if they had a relative whom they wished participated, too.

### 2.3 Data collection

FG interviews were conducted in meeting rooms at the two clinics, facilitated by the first author. We planned the FGs to last 3 hours, because we did not want to rush through the meetings, but have time enough for informal talk in a break. An open‐ended interview guide (Appendix S2) was used based on survey results^16^ and scientific literature.^1^, ^6^, ^22^, ^25^, ^26^, ^27^, ^28^, ^29^, ^32^ FGs were audiotaped and transcribed verbatim by AZ. The facilitator introduced to the overall study and presented herself as a nurse with experience in breast cancer care and with an interest in further developing the surgical breast CPP but not in direct contact with patients on a daily basis. Suggestions for change were emphasized, and it was pointed out that the aim was not to reach consensus.^20^ Subsequently, each participant made a brief self‐introduction.

The diagram for the surgical breast CPP (Figure 1) served as a framework for FG discussions and themes were prepared in advance, but participants were allowed to raise other themes. At the end of each FG, the facilitator briefly summarized the themes and invited further comments.

### 2.4 Data analysis

The overall analytic strategy was meaning condensation^33^ and systematic text condensation.^34^ The facilitator conducted a preliminary brief analysis at the end of each FG when summarizing and asking participants for comments. Shortly after each meeting, the facilitator added any written reflections about the meeting and integrated important new themes from the previous FG into the next. In the systematic analysis, the facilitator and co‐authors first read the transcripts to get an overview; this process yielded preliminary themes. Secondly, we read the text to extract specific meaning units. Coding of the meaning units during an iterative process resulted in conclusive themes (Table 1). Finally, the facilitator synthesized the transformed meaning units into a descriptive text for each FG. After having conducting all FGs, the facilitator summarized all themes and sent the condensation of the themes to patients and their relatives, allowing them to confirm the contents and provide feedback.^33^, ^34^ The patients confirmed the content with two comments, which did not add anything new to the analysis.

**Table 1:** Example of analysis process

Theme from the interview guide: How was your experience of getting the breast cancer diagnosis?		
Raw data	What did they talk about	Theme
It was a radiologist… you know he was from here [the hospital] and he told me just before I left: “I think you must prepare for a breast cancer diagnosis”. So I was prepared for the diagnosis in the next consultation. I haven't been anxious…for me it was the right person at the right time. I went back to my work and told them [the colleagues] that it probably was cancer	It was all right to get a warning about the diagnosis in the radiological clinic.	Receiving an early warning of the diagnosis in the radiological clinic (**key point**)
	Feelings about the diagnosis	How to deal with the cancer diagnosis (**coping**)
	Involving the colleagues in the diagnosis	
You walk in all alone and he says [the radiologist]… it is cancer…and then he made a biopsy…and the nurse didn't say anything…it was just bang slam. It was so terrible…so terrible. When I came outside and I was all alone…I felt so unwell and I nearly fell to the ground [the voice shows emotions]…the doctor he should…it feels like yesterday…it was terrible…and it has marked me ever since. I think there was no doubt about the diagnosis, but it was the way the message was delivered, without empathy	It was unexpected to get a diagnosis in the radiological clinic. She went to the clinic alone.	Receiving the diagnosis in the radiological clinic (**key point**)
	Feelings when receiving the diagnosis	Need related to the diagnosis (**empathy**)
	The diagnosis was delivered without empathy and has consequences for the future	The first meeting is crucial
	Lack of empathy had implications for the future	
I went to a mammography… and you know… my mother died from breast cancer…and I always went to my mammography. I had observed something at my nipple, but I didn't think about it because I have so many water cysts. Then the letter came… and then I knew something was wrong…so I brought my sister with me. The doctor [the radiologist] raised his eyebrows and said when he looked at ultrasound: “You have three water cysts…but that one I don't like…” then I knew that it was cancer. When I came to the breast clinic the surgeon asked me about family and I told about my mother and he said to me: “You know you do not die from this one”…and that have followed me all through the treatment… it gave me spirit	Knowledge about breast cancer and the need to go to mammography screening	Knowledge about breast cancer (**background**)
	Did not react on symptoms because of earlier benign cysts.	Clue about a possible breast cancer before arriving to the hospital
	The invitation for further investigation was a warning and she brought a relative to support her.	Warning of the diagnosis in the radiological clinic (**key point**)
	She had another warning about the diagnosis by facial expression and words of the radiologist	Meeting the surgeon (**key point**)
	She experienced support from the surgeon related to her earlier experience with her mother	Need related to diagnosis (**empathy and support**)
	Empathy and support from the surgeon helped her keep up the spirit through the treatment	The support was crucial

## 3 FINDINGS

**Figure 2:**
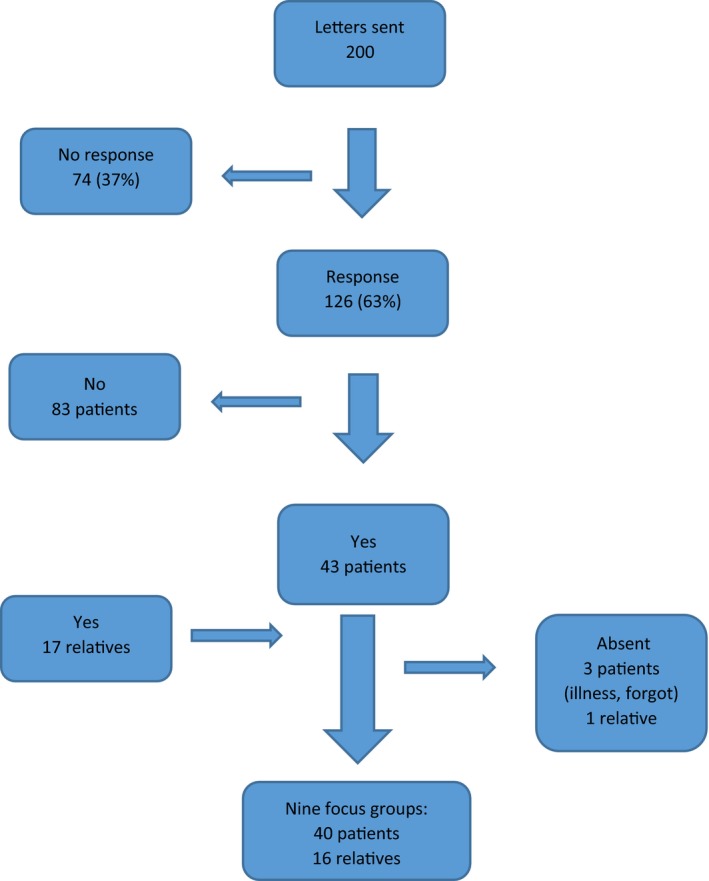
Flow Chart. Participating patients and relatives

A total of 126 patients responded to the invitation and 43 patients and 17 relatives agreed to participate; however, due to illness, only 40 patients and 16 relatives participated in nine FGs (Figure 2). Table 2 shows the participating patients’ profile and Table 3 the distribution in FGs. The time since diagnosis ranged from 8 to 18 months.

**Table 2:** Profile of the participating patients

Age	61.5 (32‐81) y
Surgery	
Mastectomy	14 (35%)
Lumpectomy	26 (65%)
Axillary clearance	16 (40%)

**Table 3:** Focus groups

	Participants	Patients	Relatives	Time (h)
FG 1	2	1	1 (male)	2
FG 2	4	3	1 (male)	3
FG 3	6	6	0	3
FG 4	7	7	0	3
FG 5	8	3	5 (4 male, 1 female)	3
FG 6	4	0	4 (male)	3
FG 7	9	8	1 (male)	3
FG 8	8	6	2 (1 male, 1 female)	3
FG 9	8	6	2 (male)	3
Total	56	40	16	26^a^

FG, Focus group.

a Breaks included but not transcribed.

### 3.1 The surgical breast CPP: a journey with several key points

Although the focus was on key points in the surgical breast CPP, patients and relatives experienced the breast CPP as a “journey” of experiences from the encounter with the health‐care system to daily life as a cancer patient and a survivor. They did not distinguish between different departments in the journey, but the facilitator recognized the different settings by asking to the context of the experience.

All participants considered the surgical breast CPP fast, predictable and well planned. They used metaphors like “a train on its tracks” and “following a thread” to describe it. All patients had undergone surgery, and the majority had received adjuvant therapy. The diagnosis was considered scaring and severe. However, the time in the surgical department was short, and most patients called this “the easy part.” Patients who received chemotherapy experienced the pathway in the oncological ward as challenging and exhausting.

All patients recognized the key points in Figure 1 as crucial. However, several patients in our study mentioned the GP consultation, the investigation in the radiology clinic and the follow‐up after surgery as key points too. They described the key points as situations where the outcome was critical for their daily life and future. In the key points, they considered a supportive relationship, empathy and good communication between the patient and the HCP as crucial.

**Figure 3:**
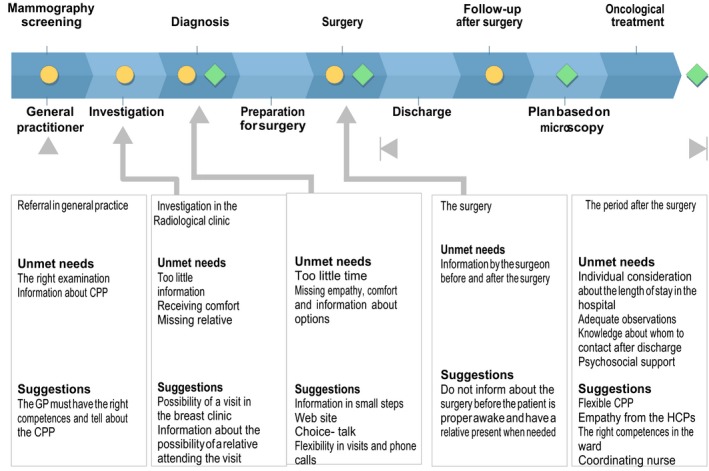
The surgical breast cancer patient pathway‐key points, unmet needs and suggestions for change from patients’ and relatives’ perspective. 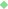 Key points from the scientific literature^1,6,22‐29^ (Figure 1). 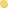 Key points from the findings

Figure 3 illustrates the key points identified in the literature complemented by patients’ and relatives’ experiences of additional key points in the surgical breast CPP and outlines their suggestions for change concerning unmet needs. In the following section, the key point will be described further. In the quotes below, we named doctors as male and nurses as female to maintain anonymity.

### 3.2 Referral in general practice

When a woman became suspicious of a lump in her breast, she would contact her GP. In the consultation, it was very important that she felt that she had the right examination and that the GP took her worries seriously. A few patients did not feel that their GP was competent performing the right examination and felt that he hesitated or refused to refer her to the breast clinic.

My doctor told me that no one of my age gets cancer… I was just a hypochondriac, and I was wasting his time…and then, after 1½ year, I had this big lump [points at her breast]… I waited too long because I didn't want to be called a hypochondriac again…I had a new doctor, and he referred me for examination and then they found that it was cancer. (Patient A43)

On the other hand, several patients found it scary that the referral from the GP to the radiological clinic was as rapid as it had been. They suggested that the GP should inform them about the CPP and explain the rapid referral.

I was stunned. Is it that serious? Later on I recognised that is was because of the inclusion in the cancer packages… my general practitioner should have told me. (Patient A78)

During cancer treatment, only few patients had contact with their GP, but those who did experienced great support in relation to their daily life and worries about the future.

### 3.3 Investigation in the radiology clinic

Some patients unexpectedly received a preliminary warning of the diagnosis by the radiologist. Arriving alone at the radiology clinic because they thought the purpose of the visit was to have a biopsy taken and that they would get the results at a later visit, they suggested that the radiology clinic recommends the patient to bring a relative. Furthermore, they needed more explanations and a caring nurse to follow up and suggested that it should be possible to see the outpatient breast clinic immediately after their visit to the radiology clinic.

You walk in all alone and he says [the radiologist]… it is cancer… and then he made a biopsy…and the nurse didn't say anything… it was just bang slam. It was so terrible… so terrible. When I came outside and I was all alone… I felt so unwell and I nearly fell to the ground [the voice shows the emotions] … the doctor he should… it feels just like yesterday… it was terrible… and it has marked me ever since. I think there was no doubt about the diagnosis, but it was the way the message was delivered, without empathy. (Patient A97)

This quote underlines the importance of the first meeting with the HCP in the hospital. The patients were sensitive of the HCP's attitude, communication and facial expressions, and a bad experience could taint coming visits to the clinic. In one of the clinics, patients were offered a short talk with a breast care nurse after their visit to the radiology clinic, which made patients feel safe.

Other patients were satisfied being warned about the diagnosis by the radiologist.

I said to him [the radiologist] … what is your experience? Tell me if you think it is cancer… and then he told me… “I think it is cancer”, he said… and it was okay because I myself had asked. (Patient B3)

### 3.4 Receiving the diagnosis and treatment plan

Receiving the diagnosis from the surgeon was an important and sensitive situation for most patients and relatives. Some patients were stunned although they had received an early warning in the radiology clinic or by the GP. Many found it difficult to cope with the situation and the large amount of information they received, and felt unable to ask questions.

You are in shock when you receive the diagnosis… I can't say it otherwise, and the way it [the diagnosis] is served… it was just flung over the table. “Yes… you have cancer and the breast needs to be removed.” I needed to recover my breath… in order to ask some questions, but he continued…”yes … there is nothing else to do” … you are so sensitive… and you need someone empathetic to be there for you and give you some comfort. (Patient A26)

Most patients wanted the “truth” about the diagnosis but pointed to the need for empathy and communication skills in those who delivered the message.

Patients and relatives had different requirements concerning information. Some wanted to know “the whole story” from the beginning; others wanted the information one piece at a time. Some suggested a website with different levels of information to supplement oral information. Others voiced a need for an extra visit or phone call to the outpatient clinic to get answers to their questions.

A few patients had difficulties understanding what the doctor told them and got confused during the consultation with the surgeon. They requested clear communication and suggested that the doctors deliver the conclusion at the beginning of the consultation.

Most patients did not experience that they had a choice of treatment and were satisfied when the surgeon made the decision. They got a shock when they had their diagnosis and felt that the doctor made the right choice.

I was very satisfied with the doctor. He told me what to do in my situation… and I rely totally on the doctors’ decisions. After all, they have an education and long experience in this field… so they must know what they are doing… and must make the decisions. I trust them [the doctors] and they must decide. (Patient A36)

However, some patients needed more information about options before deciding on treatment. In the course of their treatment, they met others who had received different surgical treatments, and wondered if they themselves had had a genuine choice of treatment. They suggested that the doctor should tell the individual patient that she had a choice, and establish whether she wanted to participate in the decision‐making process.

I did not think about it at that time [at time of diagnosis] because everything went so fast, but now it would have been nice knowing more about the options. (Patient B24)

A few patients wanted to discuss the treatment but felt that the doctor was too busy and made the choice without asking for the patient's opinion. Some patients needed time to be accustomed to their new situation to gain knowledge and to be prepared for the choice.

Although some patients experienced that it was possible to discuss the treatment, they did not have the energy or knowledge to engage in a discussion. They felt like being in a “foreign country” without a map, language skills or information about safe passages. They needed time, information and support to make the decision.

We feel like being in a foreign country … we don't know the language… we don't know how the trip will be… and we don't know what to expect. We need information and support to make decisions… and time to think it all through. (Patient A44)

### 3.5 The surgery

All patients and relatives referred to the day of surgery as an important key point and to the surgeon as an important person. Talking to him helped build a feeling of trust, at least at the time of diagnosis, and before and immediately after the surgery. They needed to hear how the surgery went from the doctor having performed the surgery.

Furthermore, many patients and relatives emphasized that a relative should be with the patient immediately after the surgery when information is delivered. The patient might still be a little dizzy and have difficulties remembering the message.

We missed the contact with the surgeon at some very important points in the treatment. He was very busy just before the surgery and had the phone under the ear while injecting the fluid… and we did not get in contact… After the surgery, I hoped to talk to the surgeon about the surgery, but he was at a meeting when I arrived … he went to my wife just after the surgery…”did he talk to me?” … my wife asked… but she [the wife] didn't remember anything… she was still very dizzy after the surgery. (Relative A88)

Other relatives found it difficult to ask questions on the patient's behalf and suggested that the surgeon should not inform about the surgery until the patient was fully awake.

### 3.6 The period after the surgery

In one of the clinics, most patients were discharged on the day of surgery, whereas patients could stay overnight in a patient hotel near the other clinic. Most patients wanted to return home as quickly as possible, but a few needed more time in the hospital and suggested introducing a choice of time of discharge. They did not feel ready to return home and missed doctors and nurses’ empathy.

I was sent home only a few hours after surgery… it was a big surgery. It lasted for nearly 6 hours… and I was vomiting and feeling unwell. The trip in the car on my way home was terrible. My husband made several breaks… the doctor came and talked to me… but he didn't see me…”You can go home now”, he said. (Patient B18)

Some patients found the pathway too rapid because it was difficult to take in the situation. Nevertheless, most patients appreciated the short course because the waiting time was difficult.

Most patients needed follow‐up after concluding their overall treatment and did not know whom to contact. They needed knowledge about easy access to the clinic and suggested that a specialist nurse should serve as a coordinator by phone. Some patients had cosmetic or physical problems, but thought it was not worth mentioning this because they were lucky to survive.

I have some difficulties with my scar… It is very tight, and it is difficult to move my arm… In the oncological clinic, they say that the scar is very nice, but to me it is very annoying… but in the oncological clinic, they don't care… or maybe they do not know… And I don't know if I am ungrateful, because after all I am alive. (Patient B16)

Several patients and relatives expressed a need for meeting other patients and relatives in a similar situation to get psychosocial support during and after treatment and expressed that the need was, to some extent, met by joining the FG. Some patients had a psychological relapse after having kept the family together and having had a hopeful spirit throughout their treatment. Thoughts about work and “a normal life” were scaring, and support from friends and family decreased over time.

## 4 DISCUSSION

Overall, patients and relatives found the structure of the surgical breast CPP satisfactory. However, they emphasized the importance of HCPs showing empathy and being good communicators. Five key points where patients had unmet needs and suggestions for changes were identified (Figure 3). Key points from patients’ and relatives’ perspectives were slightly different from those identified in the literature.^1^, ^6^, ^22^, ^25^, ^26^, ^27^, ^28^, ^29^, ^32^ The first key point concerned their GP and underlined the importance of a thorough examination and adequate information about the CPP. The second key point concerned the meeting in the radiology clinic where some patients lacked information, comfort and a relative being present. The third key point concerned the meeting with the surgeon at the time of diagnosis where the patients needed time, empathy, comfort and step‐by‐step information about options and the process of decision making, preferably with the option to have an extra consultation or phone call. The fourth key point concerned the meeting with the surgeon where the patients needed contact with the surgeon before and just after surgery, but only when fully awake, and a relative should be present. The fifth key point concerned the time after the treatment. Some patients wanted a longer stay in the hospital with close observations, and many requested information about whom to contact for support after concluding their overall treatment.

### 4.1 Comparison with existing literature

To our knowledge, the patients’ reaction to entering the CPP has not been investigated elsewhere. Urgent referral of cancer patients is associated with better survival.^35^ Thus, the GP's role is pivotal and approximately 74% of all cancer patients in Denmark are referred for further investigation by GPs.^36^ However, the number is most likely lower for breast cancer due to the breast cancer screening programme. Most patients did not see the GP during their treatment; however, those who did noted that the GPs focused on psychosocial issues rather than their treatment. A Canadian study identified challenges inhibiting the GP's involvement in the cancer pathway, including the GP's lack of up‐to‐date knowledge about cancer treatment and the experience that patients “disappear” in the cancer system.^37^ In a German study, patients described the GP as an important person in the cancer trajectory, and the majority (71%) visited their GP during cancer treatment. Knowing the patient's anamnesis, comorbidities and mental as well as social circumstances was a considerable strength.^38^ Involving the GP more in the CPP in Denmark might be beneficial in terms of psychosocial support, and this issue needs further exploration.

In the present study, many patients received a warning in the radiology clinic that they might have breast cancer, and some experienced lack of empathy, support and communication skills on the radiologist's part. Breaking bad news is a common task in the radiology clinic.^39^ However, an American study showed that 84% of the radiologists had received no training in communicating radiological results to patients, even though 92% reported doing so.^40^ In contrast to the patients in our study, cancer patients in the UK described the content of the information given to them as more important than facilitative or supportive aspects.^41^ Communicating results in a suboptimal way might have serious implications for later experiences in the course of illness and treatment,^42^ as seen in our study where patients with negative experiences were emotionally marked in a negative way during and after treatment.

All patients in our study received the diagnosis in the breast cancer clinic and experienced the diagnosis as a stressful moment. In another Danish study, more than two‐thirds of patients with breast cancer experienced moderate or severe distress at the time they got their diagnosis.^2^, ^3^ Predictors of severe stress were young age, children living at home, use of antidepressant or sedative medicine, and prior depressed emotional status. Our patients described different coping strategies. Some wanted to know “the whole story” to regain control, while others wanted information provided stepwise. A Norwegian study of coping strategies related to breast cancer describes that a “step‐by‐step” strategy is the preferred coping style because it allows patients to gradually face their reality and prepare for what might come.^4^ In our study, the women suggested an optional phone call or an extra visit to the outpatient clinic, which is in line with the recommendations of the Norwegian study. However, in the Norwegian study, the patients preferred that the nurse made the telephone call, because patients are reluctant to call and do not want to disturb.^6^

Most of our patients were satisfied with the surgeon deciding the treatment. Some would have preferred having a choice, but only a few patients experienced having a choice. In contrast to our findings, a Danish study reported that only 5% found it appropriate that the doctor decided alone in cancer treatment, and 42% wanted a shared decision with the doctor having the last word.^31^ One explanation for this may be that the respondents in that study were potential rather than actual patients. The majority of newly diagnosed breast cancer patients are distressed^2^, ^3^ and might lack the energy, courage or knowledge to decide or discuss with the doctor. The CPP in Denmark might also signal time pressure to patients before treatment. However, one new insight achieved from the FGs was that those patients who were satisfied with the surgeon making the decision of treatment at the time of diagnosis later on in the treatment course said that they would initially have preferred a shared decision.

In line with a qualitative study about coping strategies among female cancer patients,^43^ our patients talked about being in “a foreign country,” meaning that they lacked previous experience with cancer and the health‐care system. Her identity shifted from being a healthy person to being “someone with cancer.” This shift called for new coping strategies like letting go of control and seeking social support from other women with cancer, and not only from family and friends. Being in “a foreign country” might explain the lack of capacity to be involved in their treatment decision at the time of their diagnosis.

The literature about shared decision making (SDM)^44^ identifies a need that the HCP invites the patient to become involved in the decision (choice talk), informs the patient about the possible options (option talk) and finally discusses the options in the light of the patient's preferences (decision talk). Effective facilitators for SDM include explicit encouragement to ask questions, and preparing the patient for SDM, both the process and the options available.^45^ Nevertheless, in our study, preparing the patients was difficult because they received the breast cancer diagnosis and had to decide about treatment during a single consultation.

In line with our findings, an unmet need for physical and psychosocial follow‐up after concluding cancer treatment was described by 65% of cancer patients in a large Danish survey study. The majority were in need of talking with other patients in the same situation and experienced a lack of offers related to physical and psychosocial needs.^46^ Although the focus in our study was on the surgical CPP, this finding is relevant in order to identify patients with unmet needs early on in the CPP.

The findings in our study substantiate results from the Danish National Patient Satisfaction Survey regarding preferences for involvement in treatment decisions, information and responsibility for the treatment course.^16^ However, we gained further knowledge about the key points and received details and different experiences about each subject. Furthermore, the patients and relatives made suggestions for change, which may inform the development of a more differentiated surgical breast CPP.

### 4.2 Strengths and limitations

Because of the narrow scope of the investigation for the present study and the specific characteristics of the participants, we assume satisfactory information power^47^ and data saturation^20^ in FGs with patients and relatives and with patients alone. However, in the FG with only male patients, we did not get high information power or data saturation. We obtained a picture of the challenges that a male relative encounters as a spouse to a woman with breast cancer. The FGs had different number of participants. FGs with only two‐four participants limit the range of experiences, but might be more comfortable for participants than groups of 8‐10^20^.

A number of choices in the present study were related to the fact that it was the first part of an action research study aiming at involving patients with breast cancer and their relatives in the development of the surgical breast CPP. An example is that although we might have reached saturation before conducting nine FGs, we did not want to deny any invited person the possibility to participate in this patient involvement project. Another choice related to action research was inviting patients and relatives to provide feedback on the themes.

The patient characteristics were in line with those of the average population with a diagnosis of breast cancer in terms of age and method of surgery. We have no information about educational level, social income, health literacy or marital status of either participants or non‐participants. However, we excluded patients with advanced disease. Some of the non‐participants responded that they lacked energy, suggesting that the participants were healthier than those who did not participate.

The period between the FG and the patients’ and relatives’ experience of the surgical pathway was up to 18 months, during which the organization of the pathway changed a number of times. The patients helped each other remember experiences, but the retrospective nature of our study design is a challenge with respect to remembering details from experiences. Additionally, if the FGs had been conducted while the patients were still receiving treatment, the findings might have been different, because of a stressful situation at time of diagnosis,^1^ and because patients’ evaluation of care depend on where they are situated in the CPP.^48^

## 5 CONCLUSION AND IMPLICATIONS FOR PRACTICE

Patients and relatives experienced the structure of the surgical breast CPP as satisfactory and well planned. However, in relation to five perceived key points of the pathway, the patients experienced unmet needs regarding communication, information, support, comfort and choice of treatment. Furthermore, the patients requested flexibility with respect to the number of visits, the duration of stay in the hospital and access to the clinic.

Implementation of the findings calls for training of HCPs’ communication skills and more knowledge about the concept of SDM among both patients and HCPs. Flexibility in the surgical breast CPP must be possible, and the HCP's motivation for changing the daily practice is of utmost importance. Finally, the development of the surgical breast CPP based on patients and relatives’ experiences requires interdisciplinary co‐operation between HCPs and the leaders of the organization.

## CONFLICT OF INTEREST

None declared.

## ETHICS

The Danish Data Protection Agency (record number 2007‐58‐0010) approved the overall study and accepted that information from patient files was passed on to the researcher from the Danish Breast Cancer Cooperative Group database.^23^ The study obtained informed written consent from all participating patients and relatives. During the project, we negotiated further participation when relevant. The study does not fall under the jurisdiction of the Danish Health Research Act (enquiry 73/2014) and was therefore exempted from approval by the Regional Committee on Health Research Ethics.^24^ Throughout the study, we followed the ethical guidelines of the World Medical Association Declaration of Helsinki.^25^
