# Supplementary material for: Surgical breast cancer patient pathway: Experiences of patients and relatives and their unmet needs
Source: Health Expect. 2019 Jan 12;22(2):262–72. doi: 10.1111/hex.12869 (PMC6433326; doi:10.1111/hex.12869)
Supplement: Supplementary file 1 [file HEX-22-262-s001.pdf]

I wish to participate in the project:

YES ☐

I had surgery for breast cancer ____________________(date)

Name ____

Phone ____________________

E-mail ____________________

Signature

YES ☐

I am a relative to a patient who had surgery for breast cancer

________________________(date)

Name ____

Phone ____________________

E-mail ____________________

Signature

I do not wish to participate in the project because:

**Please tick off one or more reasons**	
I am not interested	
I have no time	
I have no energy	
Transportation time is too long	
The time schedule does not suit me	
I cannot contribute with anything new	
Other reasons, please describe:	

I had surgery for breast cancer ☐

I am a relative ☐

With my signature I declare that I wish to participate in the project:

Patient involvement. Development of the surgical breast cancer patient pathway.

I allow the project leader, XXXXXXXX, to contact me either by phone or by e-mail.

Whenever I wish to, I can withdraw my participation without stating the reason why

My contribution to the project will be kept anonymous.

Contact:

XXXXXXXX, Clinical Nurse Specialist, PhD student
XXX
XXX
XXX

ingrzoey@rm.dk
Phone xxx

CONSENT TO PATIENTS AND RELATIVES

Patient involvement.

Development of the surgical breast cancer patient pathway

AARHUS UNIVERSITY

Aarhus University Hospital
