# Supplementary material for: Surgical breast cancer patient pathway: Experiences of patients and relatives and their unmet needs
Source: Health Expect. 2019 Jan 12;22(2):262–72. doi: 10.1111/hex.12869 (PMC6433326; doi:10.1111/hex.12869)
Supplement: Supplementary file 2 [file HEX-22-262-s002.pdf]

Interview guide for focus groups

**Starting:**<br>Thank you for participating<br>Aim of the project<br>Confidentially, anonymity, recorder, how to proceed with the project<br>Roles, themes, breaks, transportation	
**Presentation**	
Facts and motivation	Self-presentation of each participant: name, time of and method of surgery, motivation for participating in the project
**Introduction**	
Surgical breast cancer patient pathway	Presentation of the healthcare professionals' "model" of the pathway.<br>Legal framework, evidence, experience, practical issues
**Introduction questions**	
Receiving the diagnosis	What brought you to the doctor/hospital?<br>How was your experience of getting the breast cancer diagnosis? (as a patient and as a relative)<br>How was your first meeting in the hospital?
**Transitions questions**	
Surgery for breast cancer	Do you recognise the pathway in the ”model”?<br>How was your experience of the breast cancer patient pathway?<br>What was important for you in the treatment course?<br>What was good? What was difficult?<br>Other comments?
**Key questions**	
Information	How was your experience of the information about diagnosis, surgery and the rest of the treatment?<br>Sufficient? Too much? Too little? The timing? Additional questions? Other issues?<br>Did you search for more information? What kind of information? Which sources did you make use of?<br>Other comments?
Communication	How did you experience the communication?<br>Communication between you and HCPs<br>Communication between HCPs and between different departments/hospitals?<br>Clear, agreement, continuity?<br>Other comments?

Coherence	What does coherence mean to you?<br>How was your experience of coherence in the pathway?<br>Other comments?
Individuel solution	Did you have individual needs? Were the individual needs met?<br>Was it possible to have a differentiated patient pathway to meet your needs?<br>Other comments?
Responsibility	How did you experience the division of responsibility in the treatment course?<br>Would you have preferred more or less responsibility?<br>Other comments?
Decisions	How were the decisions made?<br>About treatment (surgery options or other options)<br>Inpatient or outpatient?<br>Discharge or other decisions?<br>Other comments?
Other themes	Do you have other themes you would like to discuss?
Changes in the pathway	Do you have suggestions for changes to the breast cancer patient pathway based on your experiences?<br>Do you have any advice for future patients and relatives?<br>If you should point to an important thing to say to the HCP, what would it be?<br>Do you have any advice for the HCPs?<br>Other comments?
**Summary**	
Joint summary<br>Make a break in order to let everyone think the meeting through	What was most essential in this meeting?<br>Anything surprised you?<br>Agreement and disagreement?<br>Unexpected themes or discussions?<br>Other comments?
**Preliminary conclusion**	My preliminary conclusion: is it in in line with your experiences?<br>Do you have anything to add or themes to unfold?<br>Misunderstandings or forgotten issues?
**Closing remarks**	A written summary of all the meetings and the possibility to comment on it.<br>The possibility to participate in a dialogue meeting with healthcare professionals<br>May I contact you for further information?<br>Call or write me if anything occurs that you want to talk about.<br>Thank you very much.<br>I will keep you up to date on the project. Results from the dialogue meetings and any changes made to the pathway.

HCP: Healthcare professionals
